# 

**Published:** 1876-12

**Authors:** 


					﻿Vol.. 23.
December, i«™.
No. 12.
TWENTY-THIRD YEAR.
HALL'S
Journal of Health.
PUBLISHED AT 137 EIGHTH STREET, NEW YORK,
Four Doors East of 756 Broadway.
Copyright 1876, by E. H. Gibbs.
AGENTS FOB THE TRADE:
AMERICAN NEWS COMPANY, 121 NASSAU STREET.
NEW YORK NEWS COMPANY, 18 BEEKMAN STREET.
Terms, $ 1.50 a Year.
SINGLE NUMBER, 20 CENTS.
Jobs Kint. Printer. 11 Frankfort St.. New York.
				

